# There Is an Inverse Correlation between Basic Motor Skills and Overweight in Schoolchildren Aged 8 to 12

**DOI:** 10.3390/children8121198

**Published:** 2021-12-18

**Authors:** Lilyan Vega-Ramirez, Rosa M. Pérez-Cañaveras, Joaquín De Juan Herrero

**Affiliations:** 1 Department of General and Specific Didactics, University of Alicante, San Vicente del Raspeig s/n Road, 03690 Alicante, Spain; 2 Nursing Department, University of Alicante, 03690 Alicante, Spain; rm.perez@ua.es; 3 Department of Biotechnology, University of Alicante, 03690 Alicante, Spain; jdj@ua.es

**Keywords:** primary school, motor skills, motor learning, physical education

## Abstract

In the last three decades, childhood obesity has become a 21st century epidemic, a product of social development. The purpose of this study was to analyze the repercussions that overweight and obesity have for the basic motor skills of a group of children in primary school, as well as their interrelations. We analyzed a sample of 287 students from Spain, aged between 8 and 12 years. Anthropometric data were taken to determine their Body Mass Index (BMI). A scale of assessment of basic motor skills was used to evaluate their motor skills. The BMI data revealed that 11% of this sample was considered obese, and 26% was overweight. Children showed higher competence in locomotor skills than in object control and turn and rolling skills, for which motor competence levels were lower. Likewise, there was an inverse relationship between BMI and basic motor skills; children with obesity had the lowest levels of motor skills, and there was a significant difference regarding non-obese children (*p* ≤ 0.05). These results showed that overweight and obese children have lower basic motor skills, which can lead to the abandonment of physical activity and the preference for other activities that reinforce a sedentary lifestyle.

## 1. Introduction

Primary education aims to strengthen the social, affective, and motor cognitive development of students, and for many children this stage is the beginning of formalized and systematic activity [1,2]. One of the objectives of physical education in primary education is to develop basic motor skills. Basic motor skills are phylogenetic actions that are species-specific and play a relevant role in survival. These include displacing, jumping, turning, receiving, and throwing, which in turn are the basis for the development of other more specific skills [3,4]. From birth, physical and neurological maturation, the quality and variety of experiences, genetic and environmental conditions, quality of life, a balanced diet, and hygiene are factors that are directly involved in motor development [3,4,5,6].

A factor associated with low levels of basic motor skills is a sedentary lifestyle [7,8]. The development of motor skills is a process that requires the experience and maturation of the central nervous system [4,9,10]. In this way, the motor stages that make up the process (initial, elementary, and mature) are reached; therefore, it is vitally important that children have the opportunity to practice and experience different forms of movement with a logical and systematic approach [11]. If they do not have the opportunity to practice, these basic motor skills will not reach the mature stage, which can cause coordination development problems [4,5]. The social evolution of recent decades, in both developed and developing countries, has led to a change in human behavior. Home technology easily provides entertainment that replaces activities that were previously performed outdoors and required physical exercise. Along with a sedentary lifestyle and changes in the industrialized production of food, products with a high content of fast-absorbing fats and carbohydrates contribute to overweight and obesity, as they induce an accumulation of adipose tissue [12,13,14,15], thus increasing the number of overweight people and causing problems that harm the health of the general population [16,17,18,19,20,21].

Obesity is considered a 21st century pandemic that affects the general population; in the case of children and adolescents, its increase and prevalence are alarming and concerning for health institutions [19,22,23]. Worldwide, more than 41 million children under the age of 5 were overweight or obese in 2016 [23]. The situation is no different in Spain, where in 2015, 23.2% of children between the ages of 6 and 9 were overweight, and 18.1% were obese. Several studies indicate that overweight children and adolescents are more likely to have cardiometabolic diseases, metabolic syndrome, and alterations in musculoskeletal capacity [19,20,21]. The prevalence of such pathologies could be related to problems in the development and learning of basic motor skills. Some studies [7,8] indicate that overweight and obese children have lower levels of fundamental movements.

The objective of this study was to analyze and interpret the repercussions that overweight and obesity, expressed through the Body Mass Index (BMI), have for the basic motor skills of a group of primary school students, as well as their interrelationships.

## 2. Method

This study utilized a descriptive and quantitative cross-cutting design, including 287 school children (Table 1), aged between 8 and 12 years (average 9.97, ± 1.305), at 3 primary schools, specifically, 1 public school (CA) and 2 private schools (CC, CSS), in a province of Spain. The selection of the schools was carried out for convenience and availability. Both the students’ parents and the head of each school provided an informed consent, following the guidelines of the data projection law and the approval of the Ethics Committee of the University of Alicante (UA-2020-09-01).

### 2.1. Study Instruments

The Scale for the evaluation of basic motor skills was used [24] to measure motor competence. This scale is a dichotomous list of motor tasks that assumes that each task can be assessed with 2 alternatives, adequate or not, to determine the level of difficulty in execution by the age of the subject. The tasks to be carried out range from simple to more complex activities. In the case of the locomotive skill, the tasks range from running in a straight line for 12 m, to running without changing pace, passing with steps in a row of Swedish benches. In object control, the tasks range from throwing a ball over the head with both hands and catching it while suspended, again with both hands, to bouncing two balls consecutively with both hands 10 times with closed eyes. In the case of the turn and rolling body skills, the tasks range from a quarter turn, jumping from a high place to a knee-high plinth and falling with the feet together, to being located within a 60 cm diameter circle, completing a 360° rotation while suspended, and falling inside a hoop. The levels of difficulty are provided by the evaluation test itself (low, medium low, medium, medium high, and high) together with the achievement criteria. The tasks were presented in the same order as the prepared task list. If the task was successful, it was scored as 1; otherwise, it was scored as 0. A second attempt was allowed if the first attempt failed. When a subject failed 2 more consecutive tasks after failing a task, the test was considered failed for that subject. The basic motor tasks assessed by the test are shown in Table 2.

The reliability of the instrument was determined by Cronbach’s alpha, with the following results: locomotive skills alpha 0.81, rolling alpha 0.84, and object control alpha 0.86.

The determination of weight and size for the calculation of the body mass index (BMI) was performed using an electronic scale and a base tallimeter. To determine the levels of obesity and overweight in the sample, the BMI values obtained were compared with those in a reference distribution of the Orbegozo Foundation [25]. This research considers obesity as the ≥97th percentile and overweight as the ≥85th percentile.

To learn the sporting habits, a questionnaire from the Ministry of Health and Consumption of the Generalitat of Valencia was used, which contains 15 multiple-choice questions addressing healthy habits and a sedentary lifestyle [26].

### 2.2. Procedure

Once the parents’ authorization for their children’s participation was obtained, the questionnaire on sport habits was given to the physical education teachers for distribution to the students. The scale of assessment of basic motor skills and weight and size were evaluated by the researchers.

The data collection protocol was as follows:

1. The students were informed of the assessments to be conducted;
2. weight and size measurement were performed with the children wearing shorts and a t-shirt while being barefoot;
3. each task on the basic motor skills assessment scale was explained so that the subjects understood it;
4. tasks were presented in the same order as in the prepared task list. If the task was successful, it was scored as 1, otherwise it was scored as 0;
5. a second attempt was allowed if the first attempt failed. When a subject failed 2 additional consecutive tasks after failing a task, the test for that subject was suspended.

### 2.3. Data Analysis

The IBM SPSS 26.0 software (IBM, Armonk, NY, USA) for Windows was used for data analysis, applying descriptive statistics across cross-tables and percentages and standard deviation. The Kolmogorov–Smirnov test was applied to verify the normality of the data. For the comparison of variables, variance analysis or one-way ANOVA factor, post hoc Sheffe was also used. A significance level of *p* < 0.05 was set.

## 3. Results

The results corresponding to the prevalence of overweight and obesity indicated that the average values of the BMI increased with age in the case of boys, while for girls, there was an increase between 8 and 10 years of age, as well as a slight decrease. Table 3 shows a list of the students’ anthropometric characteristics.

The BMI data revealed that 11% of this sample was considered obese and 26% was overweight.

By segregating the sample by school and BMI, the levels of obesity were similar in the three schools, albeit slightly lower in the CC school (10.9%). Regarding overweight, we observed that the CC school had a slightly lower percentage of overweight students (21.8%) compared with the CSS (28.6%) and the CA (27.4%) schools. The percentage of students with normal BMI was similar among the schools (CC 67%, CPA61%, CSS 59%). When performing the ANOVA, no significant differences were observed among the students in the different schools.

### 3.1. Locomotive Skill Level

The locomotive skill level of the total sample of children indicated that around 70% of them had a medium to medium-high level, only 13% a high level, 13% a low average level, and 6% a low level. By segregating the sample by schools and applying the ANOVA, there were no differences between the schools.

There was a significant difference of 0.001 (*p* ≤ 0.05) regarding the BMI in relation to the locomotive skill level. This difference was observed between the groups of normal weight and overweight (0.013, *p* ≤ 0.05) and between school children with normal weight and children with obesity (0.00, *p* ≤ 0.05) (Table 4).

### 3.2. Object Control Level

Object control level involves the basic skills of throwing and receiving. Launching an object is one of the most studied skills by various researchers. This ability is also present in various recreational and sporting activities for children. Our results showed that only 3% of the children reached a high level for this skill, 28% a medium-high level, one-third of the sample (33%) a medium-level, 17% a medium-low level, and 9% a low level. When applying the ANOVA, there were no significant differences between the schools.

When segregating the sample by BMI, we noted that the obese children mostly reached very low scores in the control mobile level. The ANOVA showed a significant difference between the group with normal BMI and the overweight group (0.023 *p* ≤ 0.05). On the other hand, there was also a significant difference between the overweight group and the obese group (0.066 *p* ≤ 0.05) (Table 5).

### 3.3. Turn and Rolling Body Level

Turn and rolling body skills are basic motor skills that are present in various sporting activities. Our results showed that only 3% of the sample reached a high level in this skill, 15% a medium-high level, 42% a medium level, 24% a medium-low level, and 16% a low level. The ANOVA showed a significant difference between the children of the CC and the CSS schools (0.001, *p* ≤ 0.05) and between the children of the CC and the CA schools (0.005, *p* ≤ 0.05), as reported in Table 6.

When segregating the sample by BMI, we also noted that there was a significant difference between the group of normal-weight children and the obese group (0.002, *p* ≤ 0.05); there was also a significant difference between the overweight and obese groups (0.004, *p* ≤ 0.05) (Table 7).

## 4. Discussion

The purpose of this study was to analyze gender differences and the relationship of overweight and obesity with basic motor skills of a group of children in primary school. The children showed higher motor competence for locomotor skills than object control skills as well as turn and rolling skills, for which the motor competence levels were lower. Likewise, we found an inverse relationship between BMI and basic motor skills; children with obesity reported the lowest levels of motor skills.

Basic motor skills are actions that develop naturally and are specific to humans. They comprise the abilities to locomotor skills, jump over, turn, and of object control. They are the basis for the development of other more specific skills that are required in everyday life [3,4,17,27,28]. Children’s fundamental motor patterns develop with age and range from the simplest to the most organized and complex, which are acquired through interactions with the environment [1,4,5,6].

The descriptive analysis of the primary motor skills of our sample and the studies carried out with the same test by Montesdeoca, Cabrera, Ruiz, and Barrera [29], regarding locomotor skills, showed that the students had a medium–medium-high level in this ability. On the other hand, the control of objects and the ability to turn and roll showed medium and medium-low levels. These results are in agreement with a study by Montesdeoca et al. [29], in which the control of objects and turns presented lower scores.

Data have shown that these latter skills are more complex and that locomotor skills are present in more daily activities, favoring their development or motor level. The current sedentary lifestyle reduces the natural practice of basic motor skills [8,30]. When comparing the results between schools, we observed a significant difference in the basic motor capacity of the turn and rolling body skills between the children of the school CC and the children of the schools CSS and CA; this fact may be associated with greater participation in sporting activities of the children from the CC school.

On the other hand, a children’s daily physical activity level may be restricted by their weight status, as this can cause structural and functional limitations [8,30]. Obesity is recognized today by the World Health Organization (WHO) as a 21st century epidemic, due to its impact on morbidity and mortality and its increasing rate among the populations of all countries [16,31,32]. Our study showed a prevalence of obesity and overweight of 11% and 26%, respectively. Several studies have shown that obesity is predominant in the male gender [33,34]; these results coincide with those of our study, where a higher prevalence of obesity was observed in boys, even though the difference was not significant.

The results obtained in this work showed significant differences in locomotive skills, object control, and rolling skills among children with normal weight, overweight, and obesity; these findings coincide with other studies [35,36] showing low levels of motor skills in overweight and obese children. Locomotive activities are affected by excess weight and can cause biomechanical problems in the lower body (legs), hindering such movements [37,38]. Our data suggest that children who are obese may have more difficulties in the required tasks of orienting their feet or increasing their stride. We also noted that overweight children in our study showed problems in decision-making tasks, such as how to overcome obstacles through displacement. Gill and Hung [39] pointed out that overweight children are less effective at overcoming obstacles and have less knee flexion. This may be due to the attempt to maintain stability of the joint by maintaining rigidity [11,40]. When it comes to object control, our data showed that overweight students struggled with tasks that require manually calculated coordination, including throwing precision, strength, and proper direction, scoring poorly in this task; these data are in agreement with those of other studies carried out in children and adolescents [7,41], that relate these observations to a possible lack of experience of physical activity to support a timely motor development. In this regard, Stodden et al. [11] noted that the locomotive skills develop before the object control skills. In our case, overweight and obese students still needed to develop locomotive activities. The analysis of the ability to turn and roll showed that overweight and obese students had less ability, which may be because this skill requires a greater control of the body. We did not find studies that related the motor competence of the turn to BMI. However, these low domains of motor competence may be related to biomechanical aspects such as the flexion of the knees to lower the center of gravity and the optimal body position to execute the roll.

These results show that children who are overweight and obese, and therefore less mobile, can avoid physical activity and perform other activities that reinforce their sedentary lifestyle, resulting in a greater probability of remaining overweight or obese [27].

One of the limitations of this study is that it was carried out in only one province of Spain; therefore, it would be relevant to expand the sample, including other geographical areas. It would also be interesting to use more specific motor tests to identify the possible limitations of behavior and motor development in overweight and obese children so as to determine future actions to be taken.

## 5. Conclusions

Through the body mass index of the sample, we found that overweight and obesity has increased in recent years.

Regarding the general objective of this study, i.e., the possible interrelation between body mass index and level of basic motor skills, we found a significant relationship between the two, with the lowest levels of motor skills being present in obese and overweight children.

## Figures and Tables

**Table 1:** Distribution of the sample, according to school and gender.

School	Girls n (%)	Boys n (%)	Total n (%)
CC	44 (15.33)	57 (19.86)	101 (35.19)
CSS	57 (19.86)	34 (11.84)	91 (31.71)
CA	44 (15.33)	51 (17.77)	95 (33.10)
TOTAL	145 (50.52)	142 (49.7)	287 (100)

CC = Private School; CSS = Private School; CA = Public School.

**Table 2:** Distribution of the evaluated motor tasks by level.

Basic Motor Skills	Level	8 Years Old	9 Years Old	10 Years Old	11 and 12 Years Old
Locomotive skills	Low	0 test	2 tests	1 test	1 test
	Medium low	2 tests	3 tests	3 tests	4 tests
	Medium	3 tests	2 tests	2 tests	1 test
	Medium high	3 tests	1 test	1 test	1 test
	High	3 tests	3 tests	3 tests	3 tests
Turn and Rolling body	Low	3 tests	3 tests	5 tests	5 tests
	Medium low	3 tests	2 tests	2 tests	2 tests
	Medium	1 test	1 test	1 test	1 test
	Medium high	1 test	2 tests	1 test	1test
	High	1 test	3 tests	4 tests	4 tests
Object control	Low	2 tests	4 tests	2 tests	2 tests
	Medium low	2 tests	1 test	1 test	2 tests
	Medium	2 tests	2 tests	4 tests	2 tests
	Medium high	1 test	3 tests	1 test	1 test
	High	3 tests	2 tests	2 tests	2 tests

**Table 3:** Distribution of weight, size, and body mass index (BMI) statistics by gender and age.

	Boys							Girls						
	Weight/kg		Height/cm		BMI			Weight		Stature		BMI		
Age	mean	sd	mean	sd	mean	sd	*p* *	mean	sd	mean	sd	mean	sd	*p* *
8 years	35.5	7.6	1.36	0.05	19.0	2.90	84	28.8	2.58	1.31	0.05	16.6	1.0	46
9 years	37.1	7.9	1.37	0.06	19.5	3.05	79	37.4	7.89	1.38	0.06	19.4	3.4	73
10 years	39.2	9.8	1.41	0.07	19.4	3.46	70	42.5	9.63	1.45	0.06	20.1	3.7	74
11 years	45.7	9.5	1.46	0.08	19.6	3.06	70	45.1	8.47	1.50	0.05	19.8	3.1	60
12 years	50.6	10.9	1.55	0.09	20.7	2.98	79	45.7	10.7	1.53	0.05	19.3	4.1	54

* Percentile according to reference tables [25].

**Table 4:** Significant differences in the level of displacement between groups of BMI.

Dependent Variable	(I) BMI	(J) BMI	Mean Difference (I–J)	Typical Error	*p*
Locomotor skills level	Normal	overweight	0.416	0.14	0.013 *
		obesity	0.76	0.192	0.00 *
	overweight	normal	−0.416	0.14	0.013 *
		obesity	0.344	0.212	0.269
	obesity	normal	−0.76	0.192	0.00 *
		overweight	−0.344	0.212	0.269

* Difference mean is significant at *p* ≤ 0.05.

**Table 5:** Significant differences in the level of mobile management between groups of BMI.

Dependent Variable	(I) BMI	(J) BMI	Mean Difference (I–J)	Typical Error	*p*
Object control level	Normal	overweight	0.399	0.144	0.023 *
		obesity	0.598	0.198	0.011 *
	overweight	normal	−0.399	0.144	0.023 *
		obesity	0.2	0.219	0.066
	obesity	normal	−0.598	0.198	0.011 *
		overweight	0.2	0.219	0.066

* Difference mean is significant at *p* ≤ 0.05.

**Table 6:** Significance of the difference in the level of rolling skill between schools.

Dependent Variable	(I) School	(J) School	Mean Difference (I–J)	Typical Error	*p*
Turn and Rolling level	CC	CSS	0.669	0.143	0.000 *
		CA	0.463	0.141	0.005 *
	CSS	CC	−0.669	0.143	0.000 *
		CA	−0.207	0.145	0.363
	CA	CC	−0.463	0.141	0.005 *
		CSS	0.207	0.145	0.363

* Difference mean is significant at *p* ≤ 0.05.

**Table 7:** Significance of difference in the level of rolling skill between groups of BMI.

Dependent Variable	(I) BMI	(J) BMI	Mean Difference (I–J)	Typical Error	*p*
Turn and Rolling level	Normal	overweight	−0.008	0.138	0.999
		obesity	0.692	0.19	0.002 *
	overweight	normal	0.008	0.138	0.999
		obesity	0.699	0.21	0.004 *
	obesity	normal	−0.692	0.19	0.002 *
		overweight	−0.699	0.21	0.004 *

* The difference mean is significant at *p* ≤ 0.05.

## Data Availability

All data used to support the finding of this study are available from the corresponding author upon request. The data are not publicly available because they concern the privacy of children.
